# Automatic concept recognition using the Human Phenotype Ontology reference and test suite corpora

**DOI:** 10.1093/database/bav005

**Published:** 2015-02-27

**Authors:** Tudor Groza, Sebastian Köhler, Sandra Doelken, Nigel Collier, Anika Oellrich, Damian Smedley, Francisco M Couto, Gareth Baynam, Andreas Zankl, Peter N. Robinson

**Affiliations:** ^1^School of ITEE, The University of Queensland, St. Lucia, QLD 4072, Australia, ^2^Garvan Institute of Medical Research, Darlinghurst, Sydney, NSW 2010, Australia, ^3^Institute for Medical Genetics and Human Genetics, Charité-Universitätsmedizin Berlin, 13353 Berlin, Germany, ^4^European Bioinformatics Institute (EMBL-EBI), Wellcome Trust Genome Campus, Hinxton, Cambridge, UK, ^5^National Institute of Informatics, Hitotsubashi, Tokyo, Japan, ^6^Mouse Informatics Group, Wellcome Trust Sanger Institute, Wellcome Trust Genome Campus, Hinxton CB10 1SA, UK, ^7^LASIGE, Departamento de Informática, Faculdade de Ciências, Universidade de Lisboa, 1749-016 Lisboa, Portugal, ^8^Genetic Services of Western Australia, King Edward Memorial Hospital, WA 6008, Australia, ^9^School of Paediatrics and Child Health, University of Western Australia, WA 6008, Australia, ^10^Institute for Immunology and Infectious Diseases, Murdoch University, WA 6150, Australia, ^11^Office of Population Health, Public Health and Clinical Services Division, Western Australian Department of Health, WA 6004, Australia, ^12^Academic Department of Medical Genetics, Sydney Children’s Hospitals Network (Westmead), NSW 2145, Australia, ^13^Discipline of Genetic Medicine, Sydney Medical School, The University of Sydney, NSW 2006, Australia, ^14^Max Planck Institute for Molecular Genetics, 14195 Berlin, Germany, ^15^Institute for Bioinformatics, Department of Mathematics and Computer Science, Freie Universität Berlin, 14195 Berlin, Germany and ^16^Berlin Brandenburg Center for Regenerative Therapies, 13353 Berlin, Germany

## Abstract

Concept recognition tools rely on the availability of textual corpora to assess their performance and enable the identification of areas for improvement. Typically, corpora are developed for specific purposes, such as gene name recognition. Gene and protein name identification are longstanding goals of biomedical text mining, and therefore a number of different corpora exist. However, phenotypes only recently became an entity of interest for specialized concept recognition systems, and hardly any annotated text is available for performance testing and training. Here, we present a unique corpus, capturing text spans from 228 abstracts manually annotated with Human Phenotype Ontology (HPO) concepts and harmonized by three curators, which can be used as a reference standard for free text annotation of human phenotypes. Furthermore, we developed a test suite for standardized concept recognition error analysis, incorporating 32 different types of test cases corresponding to 2164 HPO concepts. Finally, three established phenotype concept recognizers (NCBO Annotator, OBO Annotator and Bio-LarK CR) were comprehensively evaluated, and results are reported against both the text corpus and the test suites. The gold standard and test suites corpora are available from http://bio-lark.org/hpo_res.html.

**Database URL:** http://bio-lark.org/hpo_res.html

## Introduction

The Human Phenotype Ontology (HPO) (1) is widely used for the annotation of human phenotypes and has been employed in many biomedical applications aiming to understand the phenotypic consequences of genomic variation (2). Such applications include: linking human diseases to animal models (3–5), inferring novel drug interactions (6), prioritizing gene-disease targets (7, 8) and describing rare clinical disorders (9).

Linking from the literature to conceptual systems like HPO has been an ongoing endeavour within the text mining community that attracted substantial interest, e.g. (10–12), because of its potential for exploiting the data from millions of existing patient reports, case studies or controlled trials. This concept recognition (CR) task is similar to other well-studied tasks such as gene or protein name normalization, yet it is accompanied by its own set of challenges. In general, the challenges associated with this task are: (i) ambiguity, i.e. the same term may refer to multiple different entities—e.g. ‘irregular ossification of the proximal radial metaphysis’ vs. ‘radial club hand’—radial refers to the anatomical entity radius in the former case and the anatomical coordinate radial in the latter; similarly ‘short long bones’ vs. ‘long metacarpals’—‘long’ acts as part of the name of an anatomical entity (the long bones) in the former and represents a quality in the latter; (ii) use of abbreviations—e.g. ‘segmentation defects in L4-S1’; (iii) use of metaphorical expressions—e.g. ‘bell-shaped thorax’, ‘hitchhiker thumb’, ‘bone-in-bone appearance’; (iv) use of hedging and various forms of qualifiers—e.g. ‘subtle flattening and squaring of the metacarpal heads’, ‘segmentation defects appear to affect L4-S1’; (v) complex intrinsic structure—the lexical structure of phenotype descriptions may take several forms. They may have a canonical form, i.e. a conjunction of well-defined quality-entity pairs, where entities represent, e.g. an anatomical structure in focus (e.g. thorax) and qualities denote certain characteristics of the entities (e.g. bell-shaped)—resulting in the phenotype ‘bell-shaped thorax’. On the other hand, they may also have a non-canonical form, in which entities and qualities are associated either via verbs (e.g. ‘Vertebral-segmentation defects are most severe in the cervical and thoracic regions’) or via conjunctions (e.g. ‘short and wide ribs with metaphyseal cupping’). At the same time, each component of a phenotype description may have a nested structure, as in ‘flattening, underdevelopment and squaring of the heads of the metacarpal bones, particularly at metacarpal IV bilaterally’. All these challenges, and in particular the latter three, makes the identification of the boundaries of phenotype descriptions particularly difficult.

To date there have only been a few controlled studies focused on the automated annotation and/or harmonization of phenotype concepts in the scientific literature (13–15). Critically, none of these have used gold standard representations, hence making it hard to compare performance, e.g. due to idiosyncrasies in the annotation method. Against this background, our study has three goals:

- to introduce the first HPO-specific corpus—aimed to provide a reference standard for bootstrapping community efforts in phenotype CR; by CR, we mean the identification of entities of interest in free text and their resolution to ontological concepts, as opposed to named entity recognition that focuses only on the first part.
- to provide a set of manually crafted test suites [adapted from the original idea proposed by Cohen *et al*. (16)]—aimed at covering a broader range of concept types and to act as a standardized manner to perform error analysis and
- to benchmark the landscape of existing phenotype CR systems against both this gold standard as well as against the proposed test suites.

Consequently, the contributions of this article include:

- a novel HPO annotated corpus consisting of 228 abstracts annotated by three experts, with a total of 1933 annotations (i.e. the text spans containing entities of interest and their corresponding HPO concepts) and covering 460 unique HPO concepts
- a set of 32 types of test suites comprising 2164 entries and structured according to the 21 top-level HPO phenotypic abnormalities and
- a comprehensive evaluation of three CR systems capable of annotating HPO concepts: the NCBO Annotator (17), the OBO Annotator (18) and the Bio-LarK CR (available at http://bio-lark.org).

## Materials and methods

### Corpus construction

Our data set consists of a set of 228 abstracts cited by the Online Mendelian Inheritance in Man (OMIM) database (19). OMIM is a manually curated collection of human hereditary disorders together with their suspected or confirmed genetic origins. Each abstract was chosen for its relevance to an OMIM human heritable disease. The collection was compiled with the aim to match the 44 complex dysmorphology syndromes discussed in the initial HPO study (20). These were originally selected from the list of Pubmed citations provided by OMIM at ftp://ftp.ncbi.nlm.nih.gov/repository/OMIM/pubmed_cited. All abstracts associated with a disease have been curated.

Table 1 lists the distribution of the OMIM entries in the corpus. The head of the distribution consists of a limited number of neurodevelopmental and skeletal disorders (e.g. Angelman syndrome, Branchiootorenal syndrome 1 or Brachydactyly type C), while the tail is comprised in majority by skeletal dysplasias (e.g. Brachydactyly type A2, Oculodentodigital dysplasia or Symphalangism C. S. Lewis type).

**Table 1.** Distribution of disorders associated with the the HPO gold standard corpus

Disorder (OMIM)	Count
Angelman syndrome (OMIM:105830)	56
Neurofibromatosis type II (OMIM:101000)	46
Basal cell nevus syndrome (OMIM:109400)	40
Branchiootorenal syndrome 1 (OMIM:113650)	27
Brachydactyly type C (OMIM:113100)	14
Branchiooculofacial syndrome (OMIM:113620)	13
Townes-Brocks syndrome (OMIM:107480)	11
Arthrogryposis distal type 1 (OMIM:108120)	9
Brachydactyly type A1 (OMIM:112500)	7
Popliteal pterygium syndrome (OMIM:119500)	6
Prader-Willi syndrome (OMIM:176270)	5
Arthrogryposis distal type 2B (OMIM:601680)	4
Van der Woude syndrome (OMIM:119300)	3
Neurofibromatosis type I (OMIM:162200)	3
Arthrogryposis distal type 2A (OMIM:193700)	3
Arthrogryposis distal type 5 (OMIM:108145)	2
Gordon syndrome (OMIM:114300)	2
Trismus-pseudocamptodactyly syndrome (OMIM:158300)	2
Schwannomatosis (OMIM:162091)	2
Neurofilament protein heavy polypeptide (OMIM:162230)	2
Hemifacial microsomia (OMIM:164210)	2
Symphalangism proximal cushing symphalangism (OMIM:185800)	2
Branchiootic syndrome 1 (OMIM:602588)	2
Arthrogryposis distal type 4 (OMIM:609128)	2
Acrodysostosis 1 with or without hormone resistance (OMIM:101800)	1
Arthrogryposis-like hand anomaly and sensorineural deafness (OMIM:108200)	1
Stickler syndrome type I (OMIM:108300)	1
Brachydactyly type A2 (OMIM:112600)	1
Charcot-Marie-Tooth disease demyelinating type 1B (OMIM:118200)	1
Arthrogryposis distal type 9 (OMIM:121050)	1
Arthrogryposis distal type 2E (OMIM:121070)	1
Crouzon syndrome (OMIM:123500)	1
Duane retraction syndrome 1 (OMIM:126800)	1
Multiple endocrine neoplasia type I (OMIM:131100)	1
Treacher Collins-Franceschetti syndrome (OMIM:154500)	1
Mesothelioma malignant (OMIM:156240)	1
Neurofibromatosis familial spinal (OMIM:162210)	1
Neurofibromatosis type III mixed central and peripheral (OMIM:162260)	1
Noonan syndrome 1 (OMIM:163950)	1
Oculodentodigital dysplasia (OMIM:164200)	1
Polydactyly postaxial type A1 (OMIM:174200)	1
Greig cephalopolysyndactyly syndrome (OMIM:175700)	1
Hutchinson-Gilford progeria syndrome (OMIM:176670)	1
Multiple pterygium syndrome autosomal dominant (OMIM:178110)	1
Symphalangism C. S. Lewis type (OMIM:185650)	1
Thumbs stiff with brachydactyly type A1 and developmental delay (OMIM:188201)	1
Waardenburg syndrome type 1 (OMIM:193500)	1
Williams-Beuren syndrome (OMIM:194050)	1
Diarrhea 1 secretory chloride congenital (OMIM:214700)	1
Cystic fibrosis (OMIM:219700)	1
Hydrocephalus autosomal dominant (OMIM:600256)	1
Bor-Duane hydrocephalus contiguous gene syndrome (OMIM:600257)	1
Cholesteatoma congenital (OMIM:604183)	1
Basal cell carcinoma susceptibility to 1 (OMIM:605462)	1

The listing includes the name of the OMIM disease and the number of abstracts associated with it (the Count column).

The corpus has been annotated by a team of three experts, or more concretely, by the creators of the HPO (Prof. Peter Robinson, Dr. Sebastian Köhler and Dr. Sandra Dölken). The clinical validity of the annotations has been ensured by two of the team members—i.e. Prof. Peter Robinson and Dr. Sandra Dölken—both with extensive clinical experience in human genetics. The actual annotation process has been performed in a peer-to-peer manner and consisted of two steps. Initially, two experts conjointly annotated the corpus. This was followed by a post-annotation validation phase, in which the third annotator—paired up with one of the two initial annotators—performed a consistency and completeness check.

Consistency has also been ensured via a set of annotation guidelines that dictated the form of the phenotypic concepts, their lexical boundaries and the process of handling negation. These guidelines are:

1. Phenotype concepts should only be considered if they are present in a canonical form—e.g. include ‘hypoplastic nails’ or ‘nail hypoplasia’, but not ‘nails were hypoplastic’.
2. Conjunctive terms are allowed—e.g. ‘synostosis of some carpal and tarsal bones’.
3. Subject to the type of conjunction, atomic text spans are to be annotated with the corresponding HPO concepts. For example, the text span ‘synostosis of some carpal and tarsal bones’ represents a conjunction of two phenotype concepts: HP: 0008368 (Synostosis involving tarsal bones) and HP: 0009702 (Synostosis involving the carpal bones). Since the qualifier is preceding the anatomical conjunction, the entire text span should be annotated with both HPO concepts. On the other hand, when the qualifier is succeeding the anatomical conjunction—i.e. a mirror of the previous case—the text span should be split into the corresponding atomic phenotypes. For example, ‘branchial arch, otic and renal malformations’ results in three annotations:
  - ‘branchial arch, otic and renal malformations’—HP: 0009794 (Branchial anomaly)
  - ‘otic and renal malformations’—HP: 0000598 (Abnormality of the ear)
  - ‘renal malformations’—HP: 0000792 (Abnormal renal morphology)
4. Negated phenotypes should be included—i.e. the text span ‘kidney anomalies’ in the context of ‘no kidney anomalies were found’ should be annotated with HP: 0000077 (Abnormality of the kidney). Here, the assumption was that negation can be dealt with at a different level and via other means.

As a note to point 1 mentioned earlier, non-canonical phenotypes have been excluded from the annotation process for two reasons. First, such lexical constructs are more likely to be present in clinical summaries or Electronic Health Records (EHRs) than in scientific publications. In practice, the corpus contains just a few such examples. Second, we were aware that, with the exception of Bio-LarK CR, the other CR systems are not able to handle such phenotypes, and as such we would not have been able to provide a fair basis for comparison.

### Benchmarking phenotype CR systems

A wide variety of CR systems have been previously described—most of which are aimed at a specific purpose or domain. In this context, we focus on and benchmark three annotation tools that are able to perform CR using the HPO: the NCBO Annotator (17)—an ontology-agnostic CR system, the OBO Annotator (18) and the Bio-LarK CR—both of which have been built with HPO as a direct target.

Other systems we might have applied include ConceptMapper (21), Whatizit (22), Bio/MedLee (23), Apache cTAKES (24), or the well-known MetaMap (25). These systems were, however, either difficult to access or did not provide a route to use HPO as a desired annotation outcome. The CR systems employed adopt a range of techniques but tend to avoid deep parsing and make use of a range of shallow parsing (e.g. for named entity recognition and part of speech tagging) and pattern-based techniques, supplemented with restrictions and inferences on HPO. In all cases, we have treated the systems as black boxes (i.e. off-the-shelf solutions) and hence we have no access to any degree of confidence they may have in their concept selections.

#### 

##### M1: NCBO Annotator

The NCBO Annotator is a CR system available online and as well as part of the NCBO virtual appliance. It identifies and indexes biomedical concepts in unstructured text by exploiting a range of over 300 ontologies stored in BioPortal (26)—the largest repository of biomedical ontologies. The system can be applied to all ontologies or restricted to a specified set—such as in our case, the HPO.

NCBO Annotator operates in two stages: CR and, optionally, semantic expansion. CR performs lexical matching by pooling terms and their synonyms from across the ontologies and then identifies lexical variants in free text and assigns annotations using Mgrep (27). Mgrep applies stemming as well as permutations of the word order combined with a radix-tree-search algorithm to allow for the identification of the best matches of dictionary entries to a particular text span. During semantic expansion, various rules such as transitive closure and semantic mapping using the Unified Medical Language System (UMLS) Metathesaurus are used to suggest related concepts from within and across ontologies based on extant relationships. For the purpose of our experiments, only the CR part is relevant.

##### M2: OBO Annotator

OBO Annotator (18) was designed to perform phenotype recognition of rare diseases specifically relating to patient case reports. Their method involves identifying a set of seed linguistic patterns from case reports in PubMed abstracts in a controlled search using cerebrotendinous xanthomatosis as the motivating topic. OBO Annotator proceeds through each sentence trying to exactly match each string within the seed pattern to HPO concepts using a longest match assumption. If no longest match can be found the candidate string is divided into shorter parts and matching is tried again, e.g. in the case of coordinated terms such as ‘brain and cerebellar atrophy’. Orthography, tokenization, stemming, punctuation and stop words are all handled within the system. OBO Annotator supports contextual variations using transitive closure on the concepts in the HPO hierarchy, i.e. to infer term similarity based on ancestors and descendants of each concept under consideration. More specific annotations are preferred over more general ones where there are overlapping annotations.

##### M3: Bio-LarK CR

The Bio-LarK concept recognizer has been developed as part of the SKELETOME project (28), with an initial goal of performing automatic annotation of skeletal phenotypes in patient clinical summaries. Subsequently, it was extended to enable phenotype CR using HPO. Bio-LarK CR uses an Information Retrieval approach to index and retrieve HPO concepts, combined with a series of language techniques to enable term normalization and decomposition (e.g. token lexical variation). In addition to standard CR, the system is able to decompose and align conjunctive terms (e.g. ‘short and broad fingers’ aligned to HP: 0009381—Short fingers and HP: 0001500—Broad fingers), as well as recognize and process non-canonical phenotypes, such as ‘fingers are short and broad’—which would be aligned to the same terms as in the previous example. This is realized via an efficient pattern matching approach that uses manually crafted rules over the shallow structure of the sentence. The recognition of non-canonical phenotypes is an optional feature of Bio-LarK CR and can be enabled or disabled subject to the intented use of the system.

### Experimental setup

The HPO gold standard corpus was used to assess the CR performance of the three above-listed systems. More concretely, the systems have been applied on the free text of the 228 abstracts, which resulted in an individual set of annotations. These annotations have then been aligned to the gold standard annotations using exact boundary matching. It is worth mentioning that exact boundary detection represents the default strategy for all chosen systems. Furthermore, the annotation guidelines (see earlier) explicitly impose the fine-grained decomposition of coordinations, hence overlap matching strategies are not required.

Standard evaluation metrics have been computed on the alignment results:

- Precision (P); P = TP/(TP + FP)
- Recall (R); R = TP/(TP + FN)
- F-Score—the harmonic mean of Precision and Recall; F-Score = 2*PR/ (P + R)

In the equations earlier, TP is the number of true positive annotations (i.e. the HPO concepts suggested by the system match those listed in the gold standard); FP is the number of false positive annotations (i.e. the HPO concepts suggested by the system do not match those listed in the gold standard); and FN is the number of false negative annotations (i.e. the number of text spans failed to be identified by the system).

#### Towards standardized error analysis: Introducing the HPO test suite package

The experimental setup described earlier provides a clear view on the systems’ CR performance on real data. However, since the coverage of the HPO terms is not exhaustive, it is not able to create a comprehensive picture of the systems’ strengths and weaknesses. Furthermore, as in the case of any other gold standard evaluation, error analysis is qualitative, the process of interpreting and discussing the errors being subject to the observations made by the evaluator.

Cohen *et al*. (16) have adopted the test suite methodology from software engineering and proposed a stratified approach to data sampling based on several criteria. Each criterion focuses on a set of concepts that share a particular property, such as length in tokens, presence of punctuation, coordination, etc. This leads to a framework able to characterize the strengths of the linguistic patterns used within each CR system and, moreover, to a platform that can be applied and shared to perform standardized error analysis.

Hence, as a second major contribution, following the work done by Cohen *et al.*, we propose and make available a set of 32 manually crafted criteria (or test cases) comprising 2164 entries. Each test case entry corresponds to the label of an HPO concept and was manually selected to conform with the corresponding test case. Furthermore, since the linguistic characteristics of phenotypes depend, to some extent, on the anatomical localization of the abnormality, we have structured these test suites according to the 21 top-level HPO phenotypic abnormalities.

**Figure 1.**
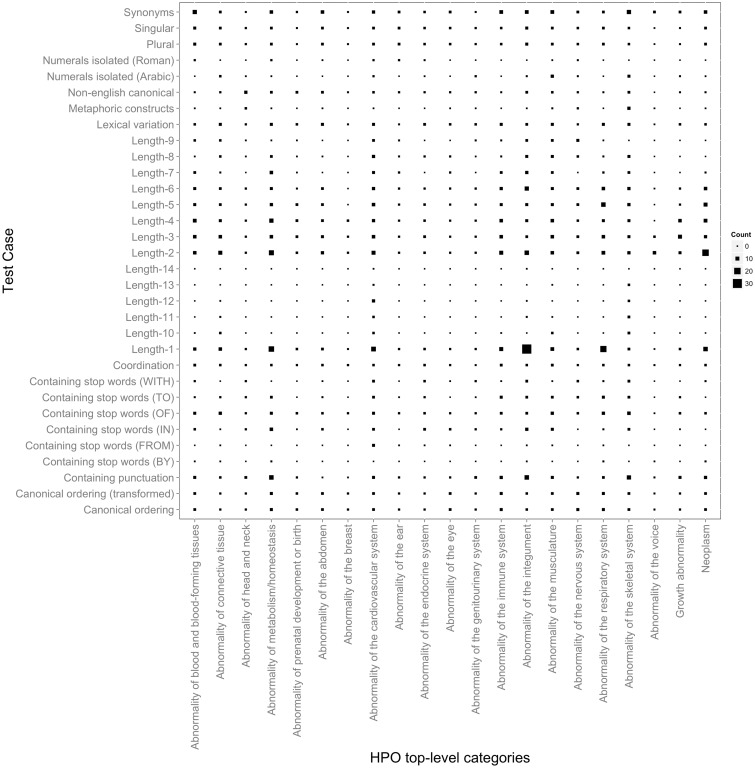
Distribution of HPO test cases according to their types mapped to the top-level HPO categories. The larger the symbol, the more test case entries the corresponding mapping has. For example, the largest number of test case entries of Length-1 is present in Abnormality of the integument. In addition to providing an overview on the test suite content, this figure also depicts a birds-eye view over the variation in terms of characteristics of the concept lexical representations in the different top-level HPO categories. We can observe, e.g. that only a very few top-level categories contain concept labels with a length greater than 10. Similarly, metaphoric constructs seem to be present only in skeletal abnormalities, which also dominate together with the abnormalities of the integument and of the metabolism the range of labels containing punctuation.

Figure 1 depicts the distribution of 32 test cases (or criteria) according to their types (their complete description is available in Section S2 in the Supplementary Material) and with respect to the 21 top-level HPO phenotypic abnormalities. On average, each criterion has 70 test case entries. The test cases can be grouped into the following categories (examples are provided in the same table):

- **Length-based tests**—characterize the system’s ability to cater for the wide variety of concept label length in tokens. The length of all HPO concept labels ranges from 1 to max. 14.
- **Tests accounting for the presence of certain types of tokens**, including punctuation, isolated numerals (Arabic or Roman) and stop words (IN, OF, TO, BY, FROM, WITH)
- **Lexical variation tests** covering the transformation of some of the tokens from singular to plural or from nouns to adjectives and vice versa
- **Token ordering tests**—opposing canonical and transformed canonical ordering
- **Synonym tests**—original concept labels are replaced by suitable synonyms listed in the ontology
- **Other, more specialized tests**, such as non-English canonical, i.e. the ability to detect non-English tokens, metaphoric constructs—phenotypes are perhaps the only domain-specific concepts that contain metaphoric expressions, e.g. bone-in-bone appearance and coordination—composite terms created via conjunctions of several atomic HPO concepts.

Figure 1 also depicts the distribution of the test cases according to the top-level HPO categories, which follows to a large extent the natural distribution of concepts in the HPO (as shown in Section S3 in the Supplementary Material). On average, each such HPO category has 100 test case entries. The actual number of entries depends on the distribution and diversity of the types of concept labels in the respective category. For example, ‘Abnormality of the voice’ and ‘Abnormality of the breast’ have the lowest coverage in HPO, and hence, also have the lowest number of test case entries assigned. On the other hand, ‘Abnormality of the cardiovascular system’ and ‘Abnormality of the integument’ have a wider variety of terms (from a lexical and morphological perspective) and hence they are better represented in terms of test cases, each with more than 150 entries. Finally, while ‘Abnormality of the skeletal system’ is by far the most dominant category in terms of coverage—almost 30% of HPO terms are under this category—this does not translate proportionally in the number of test case entries. Skeletal abnormalities have a fairly uniform lexical representation and consist of a large number of repetitions and partonomies of anatomical localizations, leading to a low lexical and morphological variety.

The test suite experimental setup was similar to the gold standard approach. We have treated each test case as an individual free text document and used them as input for the CR systems. The alignment strategy and evaluation metrics were the same as earlier, i.e. exact boundary matching with Precision, Recall and F-Score. The use of exact boundary matching in this context has the role to penalize systems that produce also concepts nested within the provided label, in addition to the concept representing the target of the test case entry. Furthemore, this is also required to enable an appropriate processing of term coordination.

## Results

### HPO gold standard corpus

The resulting corpus comprises 1933 individual annotations (with an average length of 2.42 tokens or words), which map to 460 unique concepts in HPO (4.4% coverage). In total, the annotations are related to 77 OMIM disorders.

**Figure 2.**
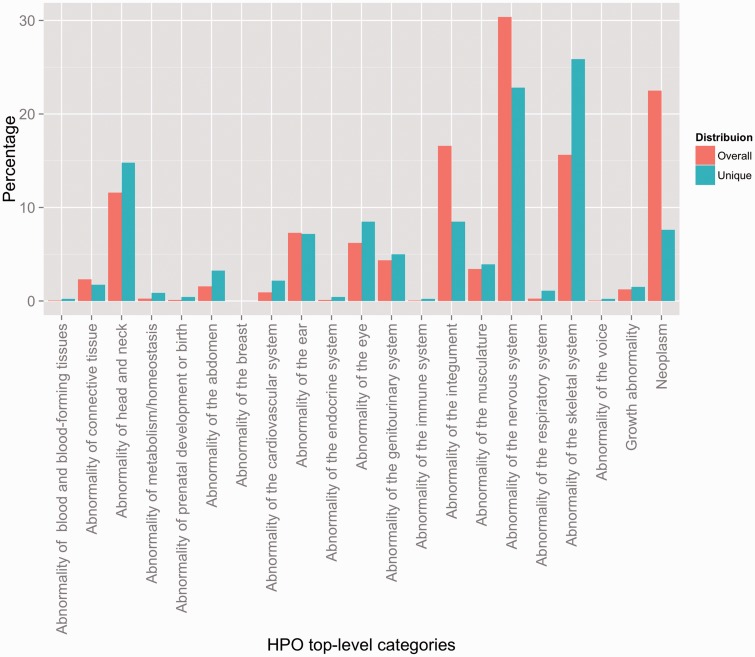
Distribution of HPO annotations according to the top-level HPO categories. Two distributions are shown: an overall distribution that accounts for duplicate concept annotations (i.e. every instance of an annotation is counted), and a unique distribution that shows the counts of the unique concept annotations (i.e. every concept is counted a single time, indifferently of how many annotations exist in the corpus).

In order to create a better overview of the concepts captured in the corpus, we have mapped them to the 21 top-level phenotype abnormalities defined by HPO. Figure 2 depicts the distribution of the resulting annotations according to these categories, when looking at both the overall percentage (i.e. counting every instance of a particular annotation—e.g. if ‘meningioma’ appears three times in an abstract, the resulting count would be three), as well as the unique coverage (i.e. counting the unique instances of each concept—using the same example, if ‘meningioma’ appears three times in an abstract, the resulting count would be one). From an overall perspective, the most highly represented concepts include abnormalities of the nervous system (30.36%), neoplasms (22.50%), abnormalities of the integument (16.6%) and abnormalities of the skeletal system (15.62%). From a unique coverage perspective, the distribution is slightly different, with abnormalities of the skeletal system, abnormalities of the nervous system and abnormalities of the head and neck dominating the corpus—25.86%, 22.82% and 14.78%, respectively. The two distributions mirror fairly closely the natural distribution of HPO concepts in the ontology (see Section S3 in the Supplementary Material).

### Experimental results

**Figure 3.**
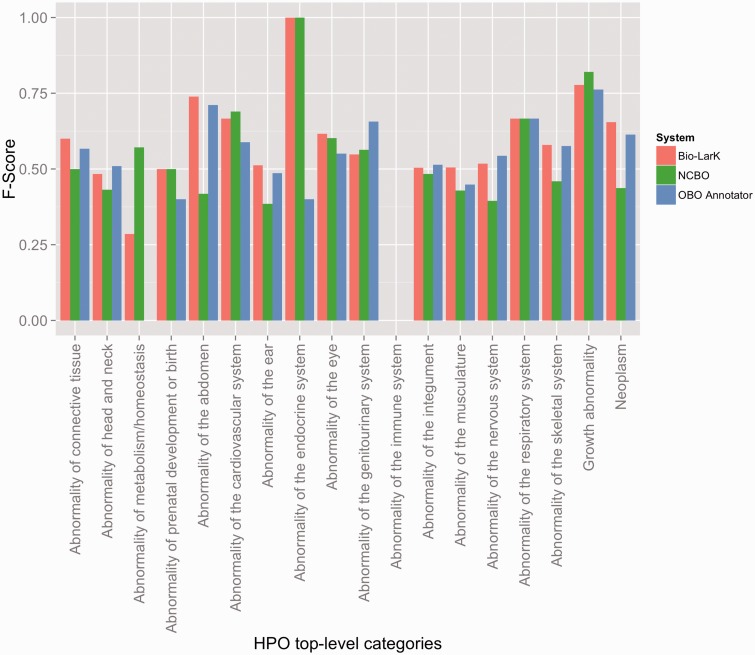
F-Score results achieved by the three systems on the HPO gold standard, distributed according to the HPO top-level category.

The evaluation results on the HPO gold standard corpus (HPO GS) are presented in Table 2, while a more detailed overview according to the top-level HPO categories is depicted in Figure 3. The OBO Annotator and Bio-LarK CR share the best performance—0.54 and 0.56 F-Score. While the overall F-Score shows minor differences, we can observe that the OBO Annotator focuses more on precision (0.69 as opposed to 0.65 of Bio-LarK) and Bio-LarK CR more on recall (0.49 as opposed to 0.44 of the OBO Annotator). The Recall of the NCBO Annotator was similar to that of the OBO Annotator—with a slight decrease of 0.05—however, the Precision, and hence the overall F-Score, was substantially lower: 0.54 Precision (on average 0.13 less the other two systems), leading to 0.45 F-Score (0.09 lower than OBO Annotator and 0.11 lower than Bio-LarK CR).

**Table 2.** System performance on the HPO corpus using exact matching and concept identification

	Precision	Recall	F1
NCBO Annotator	0.54	0.39	0.45
OBO Annotator	0.69	0.44	0.54
Bio-LarK CR	0.65	0.49	0.56

OBO Annotator and Bio-LarK CR have a similar overall efficiency, the difference in F-Score being of only 0.02. The efficiency of the NCBO Annotator was on average with 10 percentage points lower than of the other two systems.

**Figure 4.**
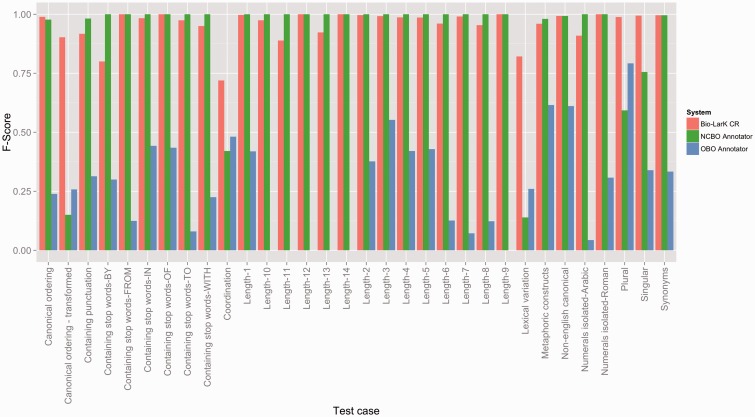
F-Score results achieved by the three systems on the HPO test suites, distributed according to the type of the test case.

Although the HPO GS evaluation has shown relatively consistent performance across all three tools, the overall results on the test suite evaluation were remarkably different—as listed in Table 3 (Figure 4 depicts an in-depth perspective according to each test case). Here, Bio-LarK CR outperformed both NCBO Annotator and OBO Annotator, achieving 0.97 Precision and an overall 0.95 F-Score. Surprisingly, the OBO Annotator, which performed on par with Bio-LarK on the gold standard, had the lowest Precision (0.54) and a very low Recall of 0.26, leading to 0.35 F-Score. Moreover, the NCBO Annotator achieved remarkable results in comparison to its performance on the HPO GS, with a 0.95 Precision (very similar to Bio-LarK)—i.e. an increase of 0.41 when compared to the HPO GS results, and a Recall of 0.84—0.09 lower than Bio-LarK, however, 0.45 higher than its Recall on HPO GS. Whilst the results of the two experiments are not directly comparable, they can be used to provide a complementary understanding of the system performance, given that one has been performed on a real-world corpus, and the other on a controlled test suite.

**Table 3.** System performance on the HPO test suites using exact matching and concept identification

	Precision	Recall	F1
NCBO Annotator	0.95	0.84	0.89
OBO Annotator	0.54	0.26	0.35
Bio-LarK CR	0.97	0.93	0.95

As opposed to the results listed in Table 2, the NCBO Annotator achieved an overall F-Score similar to the one of Bio-LarK CR—i.e. 0.89 compared to 0.95. Surprisingly, the OBO Annotator’s efficiency was much lower than of the other two systems (F1 of 0.35), although on real data it performed on par with Bio-LarK CR.

## Discussion

### Distribution of concepts in the HPO GS corpus

As mentioned earlier, Figure 2 depicts the distribution of the concept annotations according to the top-level HPO categories both from the overall as well as from the unique perspectives. It is important to remark that the interpretation of these distributions should take into account two inter-weaved aspects. First, the Overall distribution quantifies the amount of duplicate annotations in comparison to unique annotations—or more concretely the comparison between counting a particular concept annotation every time it appears against counting it only once. For example, we can observe that neoplastic, neural and integumental anomalies contain a large number of duplicate annotations—visible in the change of level from Overall to Unique. This leads to them covering only a fraction of the existing set of HPO concepts in the corresponding top-level category, while annotations of skeletal abnormalities, e.g. are in most cases unique. Second, both distributions capture the intrinsic multi-inheritance nature of the phenotypes in HPO—i.e. the fact that a phenotype concept may have ancestors leading to several different top-level categories. To be more precise, 19.12% of the set of unique concepts have more than one parent. For example, HP: 0000250 (Dense calvaria) is both an Abnormality of the skeletal system (via HP: 0002683—Abnormality of the calvaria) as well as an Abnormality of the head and neck (via HP: 0004330—Increased skull ossification). Hence, while from a unique counting perspective annotations of skeletal abnormalities are dominant, in practice, they are shared with other categories, such as abornormalities of the nervous system. In conclusion, the corpus focuses on a set of cohensive and tightly coupled abnormalities of the skeletal (including head and neck), integument and nervous systems with relations to neoplasm.

### Error analysis

The systems’ overall performance on the gold standard to some extent matched our expectations. Phenotype concepts are highly complex, in particular due to their intrinsic lexical structure, as well as due to their structural and semantic ambiguity—the reported performance reflects this complexity.

In order to provide a finer-grained view on this performance, Figure 3 depicts the F-Score achieved by the systems according to the top-level HPO category. First, we can observe that three HPO top-level categories are not present in the CR results—i.e. ‘Abnormality of blood and blood-forming tissues’, ‘Abnormality of the breast’ and ‘Abnormality of the voice’—whilst the two others have poor F-Score results—i.e. Abnormality of immune system and of metabolism/homeostasis. These results are, however, more likely due to their very poor representation in the HPO GS. On the other hand, the systems performed better on some other poorly represented categories, such as ‘Growth abnormality’, ‘Abnormality of the respiratory system’ or ‘Abnormality of prenatal development or birth’.

Second, we can use this overview as an indication of the systems’ strengths and weaknesses in recognition of certain types of HPO concepts. For example, Bio-LarK outperformed the other systems on ‘Abnormality of the abdomen’,’Abnormality of the connective tissue’ and ‘Neoplasm’, the OBO Annotator on ‘Abnormality of the genitourinary system’ and ‘Abnormality of the nervous system’, while the NCBO Annotator on ‘Growth abnormality’ and ‘Abnormality of the cardiovascular system’. No system consistently outperformed the others on all categories.

A closer inspection of the errors in the CR process reveals three particular types:

- Coordination—e.g. ‘aplastic or hypoplastic nails’—the OBO Annotator and Bio-LarK were able to correctly identify and decompose only some examples of coordination. In particular, the OBO Annotator was unable to decompose most coordinations created using the conjunctive connector ‘OR’. The NCBO Annotator identified only the tail units within a coordination (i.e. ‘hypoplastic nails’), without being able to correctly identify any entire coordination.
- Canonical order transformed—e.g. ‘Brachydactyly type A1’ instead of ‘Type A1 brachydactyly’ (HP: 0009371). Both the OBO Annotator and the NCBO Annotator were unable to correctly identify concepts that had their canonical order transformed in the text.
- Acronyms and complex concept conjunctions—e.g. ‘BDA1’ (standing for ‘Type A1 brachydactyly’) or ‘stereotyped jerky movements’—which is a conjunction of HP: 0000733 (Stereotypical motor behaviours) and HP: 0007087 (Involuntary jerking movements). No system was able to identify such concepts: (i) acronyms—because none of the systems performs acronym expansion and these were not listed as synonyms in the concept definition; (ii) complex conjunctions—these require human interpretation in order to correctly align the corresponding underlying concepts.

The above listed errors types are partly confirmed also by the test suite evaluation. Figure 4 depicts the F-Score results according to the test criteria. For example, the pair {Canonical ordering—Canonical ordering—transformed} should present mirrored results if a system is able to cater for lexical groundings that do not respect the token order as defined in the concept label (i.e. mapping ‘Hypoplasia of the optic nerve’ to ‘Optic nerve hypoplasia’). Here, we can see an important decrease in F-Score for the NCBO Annotator and only a slight decrease for Bio-LarK, which confirms the finding earlier. The OBO Annotator, has however an unexpected behaviour, reporting an increase in F-Score in this category. When inspecting the actual concepts, we were not able to find a correlation between the concept correctly identified in the canonical ordering and those in the canonical ordering transformed. More concretely, in most cases the system has identified one, but not the other, with a slight preference for the canonical ordering transformed.

The results in the Canonical ordering pair can be correlated with those in the Lexical variation category and in the {Singular—Plural} test pair (designed in the same manner as the Canonical ordering pair). Lexical variation refers to altering the lexical form of some of the tokens without altering the overall semantics—e.g. from ‘Hypoplasia of the optic nerve’ to ‘Hypoplastic optic nerve’. It is important to note that there are cases in which transforming the canonical ordering requires a certain degree of lexical variation. Here, only Bio-LarK is able to perform consistently—achieving high F-Scores (over 0.75). The NCBO Annotator has difficulties, in particular, in dealing with the lexical variation—as opposed to the OBO Annotator, which achieves an F-Score of 0.25. The same counterbalanced results are visible also on the {Singular—Plural} test case where the NCBO Annotator displays a 0.2 decrease in F-Score from singular to plural, while the OBO Annotator decreases from 0.8 F-Score on plural to 0.3 on singular—hence showing a clear preference for plural terms. Finally, the Coordination test case mirrors the gold standard results, with Bio-LarK and the OBO Annotator outperforming the NCBO Annotator.

An overall summary of lessons learned from the test suite evaluations is listed later:

- NCBO Annotator performs best when the text spans of interest map perfectly to the lexical groundings of the associated concepts—indifferently of the length of these lexical groundings or their internal composition (i.e. punctuation or stop words)
- The OBO Annotator is able to handle better lexical variation and coordination, but encounters major issues when targeting lexical groundings with higher number of tokens or those consisting of arabic numerals.
- Bio-LarK performs fairly consistently across all tests, with some difficulties with coordination and lexical variation.

Section S4 in the Supplementary Material provides additional insight into the systems’ performance on test suites, by looking at an orthogonal dimension—i.e. evaluation results according to the HPO top-level category.

It is notable that the distribution of the HPO gold standard phenotypes mirrors the categories of disorders included in the corpus—i.e. neurodevelopmental and skeletal disorders—although they also mirror to a large extent the natural distribution of phenotypes in HPO (as shown in Section S3 in the Supplementary Material). This may influence the global preception ofthe CR efficiency of a particular system. Hence, the system CR results discussed earlier should be stricly interpreted in the context provided by the corpus annotations and the possible bias towards abnormalities of the integument and of the nervous and skeletal systems (including head and neck).

Alternatively, this bias may prove the utility of the test suite corpora and offer the explanation for the large discrepancy in CR efficiency shown by the OBO Annotator on the gold standard vs. the test suite. More concretely, the OBO Annotator seems to cater well for this particular distribution of phenotypes, but cannot handle the wider range—in terms of constructs and lexical variety—captured by the test suites. Oppositely, the consistent behaviour exhibited by the NCBO Annotator and Bio-LarK CR on both corpora leads to the conclusion that they have a higher chance of achieving the same results on a new corpus—perhaps built using the same underlying format (i.e. publication abstracts).

### The goal and utility of the test suite corpus

The goal of the test suites introduced in this article is to provide a standardized benchmarking environment for error analysis—one that enables us, and others, to lay out a quantitative perspective on a set of errors a CR system may produce. Ideally, these should be a mixture of ‘standard’ cases (i.e. cases one would expect a system to handle correctly—similarly to paradigm employed in software engineering) and ‘challenging’ cases. And while we have tried to include some of the latter (e.g. the canonical order transformed or the term coordination)—the vast majority of our test cases fit into the former category. As a side remark, research on building challenging test cases in the CR context is almost inexistent. And whilst this idea has been raised, there is a paucity of published research in the area, and only lately it has started to gain some attention, in particular with the goal of building such test cases automatically (29).

By providing an evaluation both on real data, as well as on structured test cases, our intention is to describe two dimensions of the same story—dimensions that are complementary but not necessarily nested within each other. The results on the real data reflect the systems’ efficiency in the context of the concept distributions and characteristics underpinning this data. The real data covers aspects absent from the test cases and found, in their majority, in the qualitative error analysis accompanying the corresponding evaluation—e.g. lack of synonyms, lack of acronym expansion, particular forms of term coordination. The structured test cases, on the other hand—as mentioned earlier—depict a ‘what-if’ type of analysis—i.e. ‘what would be the behaviour of system X if the real data would contain a concept found in the Y test category?’ And we believe that this goal has been attained—even if more emphasis should be placed on challenging test cases. For example, the test suites show that some systems encounter difficulties in handling concepts with long lexical groundings—e.g. the OBO Annotator efficiency is inexistent for lengths greater than nine. Such a finding would not be possible with the real data, where the concepts’ average length in tokens is 2.42. Similarly, the same test suites show that the NCBO Annotator is not able to handle coordination and transformed canonical ordering, which again, is covered only in part by the real data.

In conclusion, the two dimensions require a tighter integration, in order to enable a correlation of the experimental results emerging from them. We intend to work on this aspect in the near future, in parallel to supporting the progress of the research on CR test suites.

## Conclusions and future work

Phenotype recognition is essential for interpreting the evidence about human diseases in clinical records and the scientific literature. In this article, we have presented the first corpus of manually annotated abstracts using the HPO. The corpus represents a valuable resource for gaining a deeper understanding of the linguistic characteristics of phenotypes both from an overall perspective, and with respect to their classification according to the HPO top-level categories.

Furthermore, inspired by the work of Cohen *et al*. (16), we have provided a set of 32 manually crafted HPO-based test suites. The discussion presented on the experimental results shows the utility and added-value of CR test suites. First, they provide a controlled environment for detecting patterns of errors emerging from the CR process. Identifying such patterns is beneficial for both the users of the system—as they understand the systems’ strengths and weaknesses—as well as for the systems’ developers—as they are able to focus their attention on correcting those aspects associated with the low-performing test suites. Second, test suites enable reproducibility, standardized error analysis and a fair ground for comparing system performance.

Finally, we evaluated three off-the-shelf CR systems that are able to identify HPO concepts. These have been benchmarked using both the gold standard and the test suite corpora.

Future work will focus on expanding the gold standard corpus to increase the coverage of HPO concepts and on designing additional test suites. In addition, we intend to investigate the opportunity of devising hybrid CR system configurations (or ensemble learners) that are able to take advantange the strengths and weaknesses of the individual systems, as detected by the test suites presented in this article.

## Supplementary Data

Supplementary data are available at *Database* Online.

*Conflict of interest*. None declared.

## Funding

T.G. gratefully acknowledges the support by the Australian Research Council Discovery Early Career Researcher Award (ARC DECRA DE120100508). N.C. gratefully acknowledges the support of the Marie Curie (PI: Phenominer, grant number 301806). A.O. and D.S. kindly acknowledge the support by the Wellcome Trust grant (098051) and National Institutes of Health grant (NIH) (1 U54 HG006370-01). F.M.C. gratefully acknowledges the support by the Portuguese FCT through the LASIGE Strategic Project (PEst-OE/EEI/UI0408/2014) and SOMER project (PTDC/EIA-EIA/119119/2010). G.B. acknowledges the RD-Connect FP7 grant (EU: 205444 RD-Connect), the corresponding NHMRC-EU grant (NHMRC: APP1055319) and the Raine Clinician Research Fellowship (201401). P.N.R. acknowledges the supported by the Bundesministerium für Bildung und Forschung (BMBF 0313911). Funding for open charge: Australian Research Council - Discovery Early Career Researcher Award (DE120100508).
